# Hybrid Repair of Kommerell Diverticulum and Aberrant Subclavian Artery with Compressive Symptoms and a New Strategy: Case Report

**DOI:** 10.3400/avd.cr.20-00129

**Published:** 2021-03-25

**Authors:** Junji Tsukagoshi, Yutaka Iba, Yoshihiko Kurimoto, Ryushi Maruyama, Yosuke Yanase, Naritomo Nishioka, Takahiko Masuda, Akira Yamada

**Affiliations:** 1 Department of Cardiovascular Surgery, Teine Keijinkai Hospital, Sapporo, Hokkaido, Japan

**Keywords:** Kommerell diverticulum, hybrid repair, dysphagia

## Abstract

Surgical indication and treatment for patients with Kommerell diverticulum and aberrant subclavian artery are still not well established. A patient with esophageal and tracheal compression resulting from these anatomical abnormalities was successfully treated with a hybrid approach of total arch replacement, frozen elephant trunk technique, aberrant left subclavian artery transection, and left subclavian artery reconstruction through median sternotomy. Compressive symptoms were relieved without resecting the enlarged diverticulum. In this case, the importance of preoperative investigation for the main cause of compressive symptoms is illustrated and a novel treatment strategy is outlined.

## Introduction

Kommerell diverticulum (KD) is an aortic diverticulum found at the proximal descending aorta of both left and right arch configuration that gives rise to a contralateral aberrant subclavian artery.^1)^ Named after Dr. Burckhard Kommerell who first diagnosed this abnormality in a living patient in 1936, it is a persistent remnant of the fourth primitive dorsal arch due to failed regression.^2,3)^ Prevalence for the right aortic arch (RAA) with the aberrant left subclavian artery (ALSA) is 0.04%–0.4% of the population.^1)^ Approximately 20%–60% of individuals with an aberrant subclavian artery develop KD.^1)^ Generally, 5% of adults with KD and aberrant subclavian artery are symptomatic.^1)^ Regarding its operative indication, the compressive symptoms of neighboring structures, most commonly esophagus causing dysphagia, have gained consensus.^1,3)^ For asymptomatic patients, rapid-growing lesions or the diverticulum orifice over 30–50 mm seems to be a frequently used standard; however, we should be aware of the differences in measuring the methods used.^1,4)^ Various surgical approaches and techniques have been reported, but optimal treatment is yet to be established.^1,3–9)^ We report a case of symptomatic KD, RAA, and ALSA successfully treated with a radical, hybrid approach using a total arch replacement (TAR), frozen elephant trunk (FET) technique, ALSA transection, and left subclavian artery (LSA) reconstruction.

## Case Report

**Figure 1:**
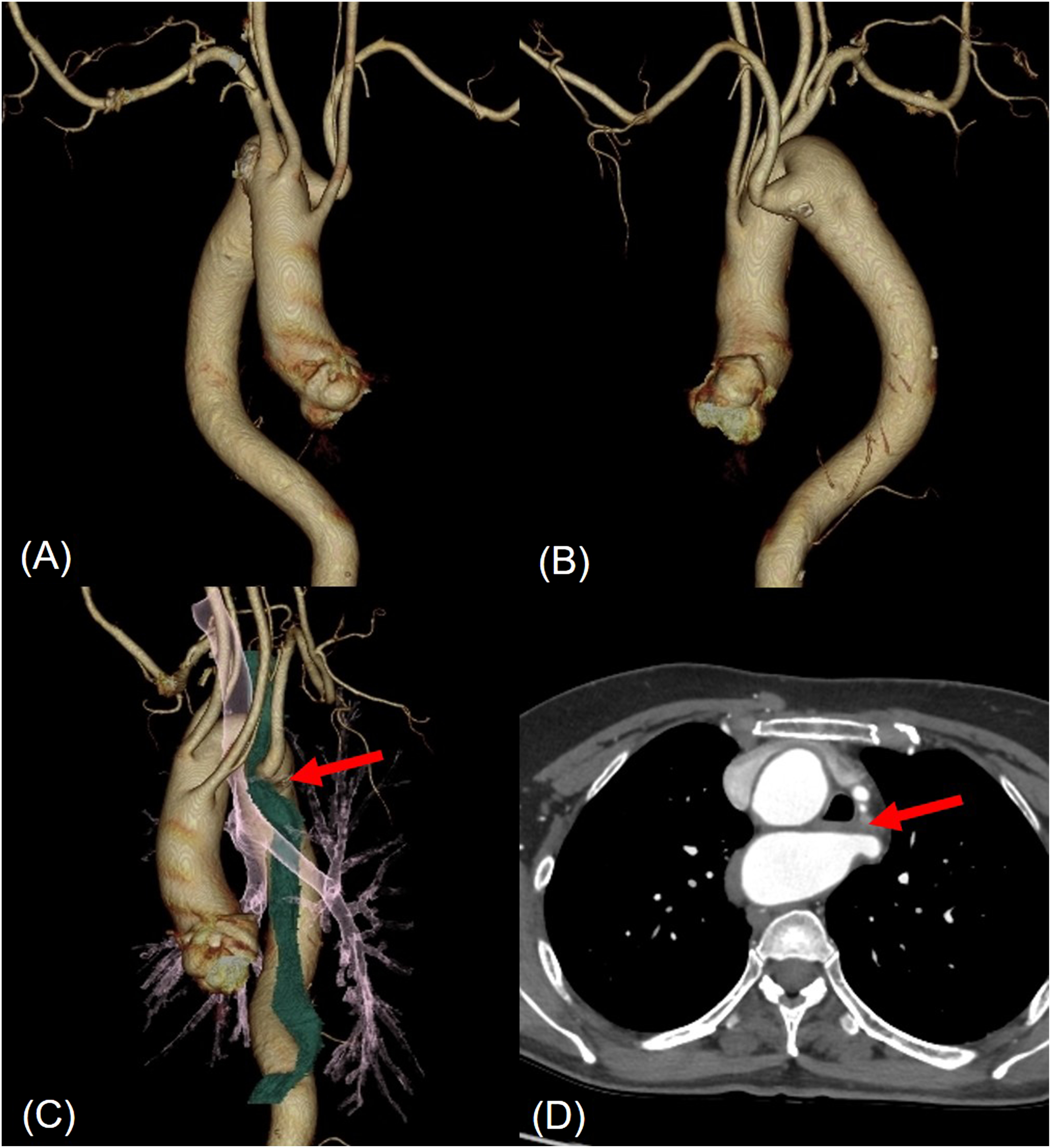
Fig. 1 Preoperative volume rendering computed tomography (CT) image of thoracic aorta. (**A**) Frontal view. (**B**) Left lateral posterior view. (**C**) Left lateral frontal view of the reconstructed three-dimensional CT showing the compressed esophagus (red arrow). (**D**) Transverse CT image of the aberrant left subclavian artery compressing the esophagus (red arrow).

A 70-year-old woman on a regular follow-up for KD, RAA, and ALSA showed a dilatation of KD orifice from 27 to 29 mm on her latest computed tomography (CT) image (**Figs. 1A** and **1B**). She was followed up for bronchial asthma diagnosed at the age of 56. A thorough interview revealed mild dysphagia from 2 years prior. Considering diverticular dilatation and compression on the esophagus and possibly trachea, a surgical treatment plan was devised. Reconstructed three-dimensional CT revealed the retroesophageal course of ALSA and the compressed esophagus (**Figs. 1C** and **1D**). Because of KD’s relatively small size and lack of symptomatic deterioration despite its dilatation, we determined that transecting ALSA adjacent to the esophagus and decompressing the KD lumen by occluding its orifice with a stent graft are sufficient for symptom relief. Given the poor proximal landing zone due to the close distance of the KD orifice to the steeply transitioning aorta from the arch to the descending aorta, we deemed thoracic endovascular aortic repair (TEVAR) unsuitable for this case. We opted for ALSA transection and LSA reconstruction along with TAR and FET.

**Figure 2:**
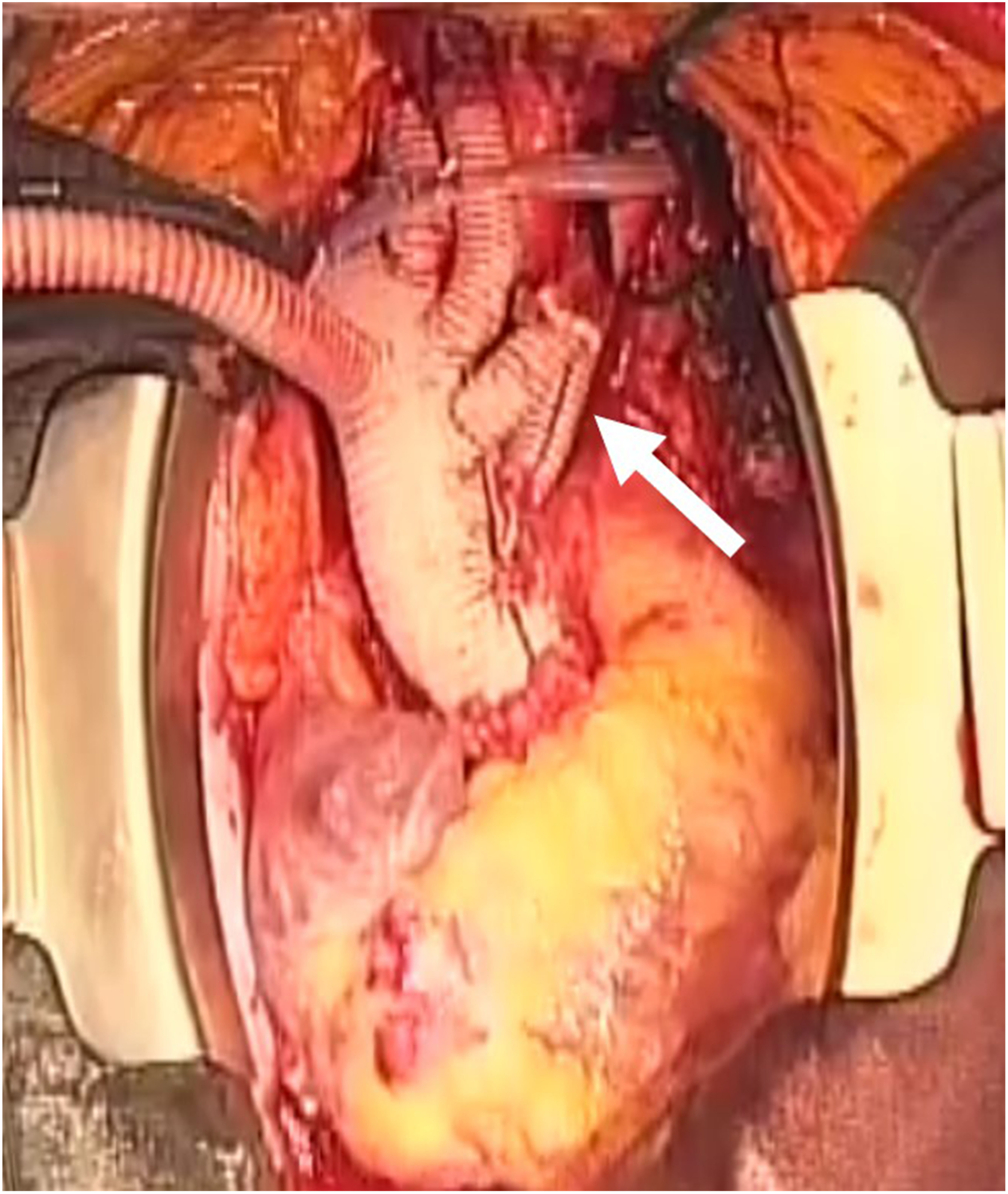
Fig. 2 Intraoperative image of the aortic arch and supra-aortic branch after total arch replacement. Left subclavian artery was reconstructed in front of the esophagus and trachea (white arrow).

**Figure 3:**
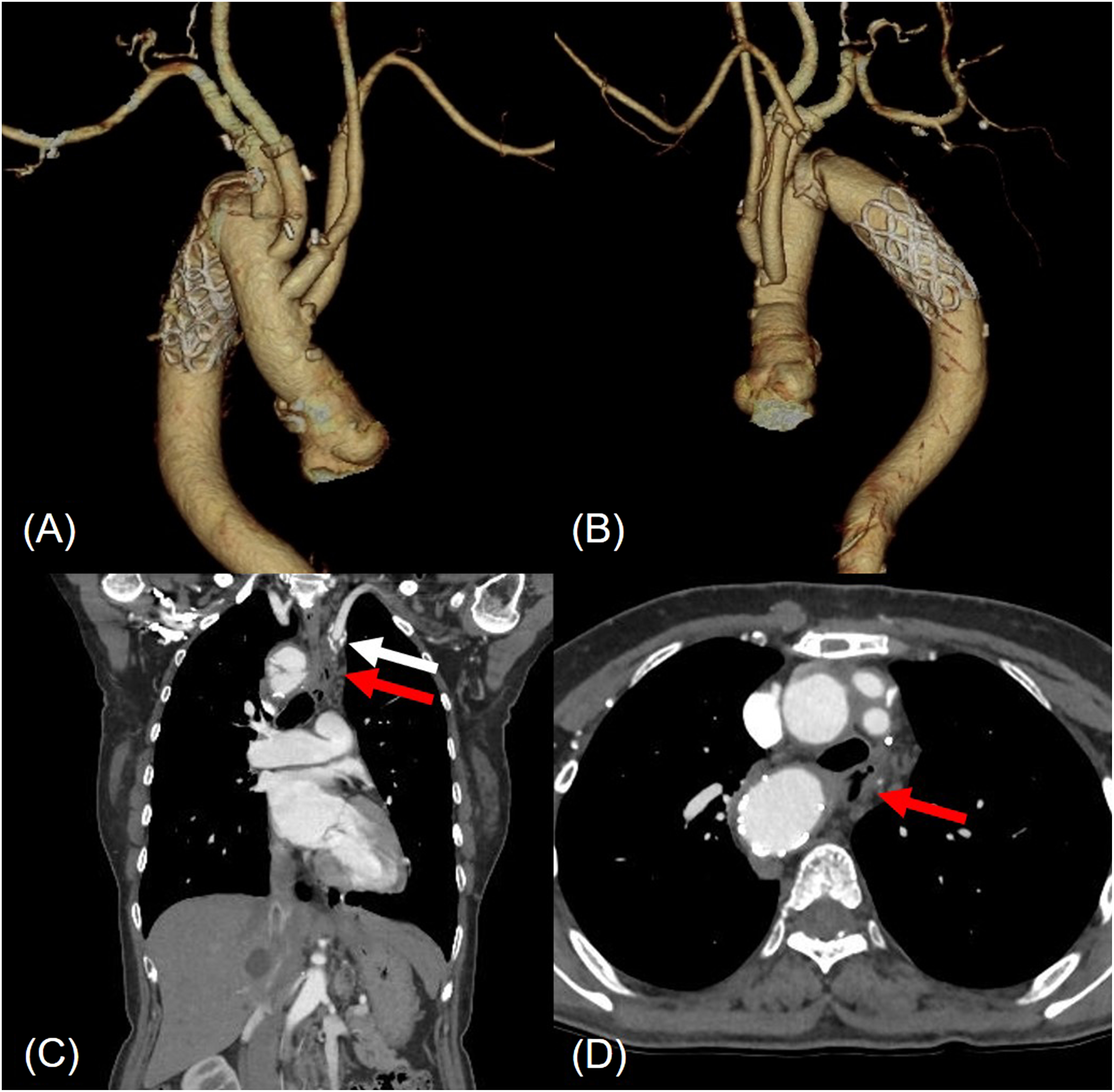
Fig. 3 Postoperative volume rendering computed tomography (CT) image of thoracic aorta. (**A**) Frontal view. (**B**) Left lateral posterior view. (**C**) Coronal CT image of the decompressed esophagus (red arrow) and the reconstructed left subclavian artery (white arrow). (**D**) Transverse CT image of the decompressed esophagus (red arrow).

Cardiopulmonary bypass (CPB) was initiated through median sternotomy with an arterial cannula in the ascending aorta, bicaval venous cannulas, integrated cardioplegia, and left ventricular vent from the right upper pulmonary vein. Systemic cooling was induced, ALSA was dissected on the left side of the esophagus and was clamped, and an 8 mm Gelweave graft (Vascutek Terumo, Renfrewshire, Scotland) was sutured to the distal segment in an end-to-side manner. Moderate hypothermic circulatory arrest at 28°C was achieved, and selective cerebral perfusion was started. The arch was dissected with extra precaution to preserve the recurrent laryngeal nerve. The distal arch was transected, and FET (J Graft FROZENIX 25×60 mm, Japan Lifeline, Tokyo, Japan) was deployed in the descending aorta to cover the KD orifice. Arch reconstruction was subsequently performed with a four-branch graft (Gelweave 22×10 × 8×8 mm, Vascutek Terumo, Renfrewshire, Scotland). It was followed by ALSA transection adjacent to the esophagus and proximal to the previous graft suture site, and its ends were oversewn. LSA was reconstructed by anastomosing the graft to the newly constructed arch (**Fig. 2**). After successful weaning from CPB and hemostasis, closure was conducted in the usual manner. The patient’s postoperative course was uneventful. Dysphagia and respiratory symptoms disappeared completely. Postoperative CT revealed no endoleaks. The thrombotic occlusion of KD, shrunken KD orifice from 27 to 24 mm, and the decompressed esophagus were confirmed (**Fig. 3**). She was discharged home on postoperative day 14.

## Discussion

Numerous surgical methods for compressive symptoms have been reported. For pediatric population, Backer et al. recommended ligamentum arteriosum release as the first choice, followed by KD resection, and then ALSA transection and LSA reconstruction if symptoms persist.^4)^ However, in adults, KD resection appears to be the most common treatment approach.^5)^ Vinnakota et al. recommended KD resection with or without interposition graft of the aorta and LSA reconstruction as the standard approach for symptomatic adult patients.^3)^

An increasing number of endovascular approaches have been reported for symptomatic KD and aberrant subclavian artery. Their effectiveness, however, is highly controversial. Several studies reported a higher failure rate of symptomatic relief with endovascular occlusion and concluded open resection of KD to be the standard for the symptomatic condition.^3,5)^

In our case, despite not performing KD resection, symptomatic relief was successfully achieved by transecting ALSA. A limited number of reports have previously described symptom disappearance in symptomatic KD cases specifically with ALSA transection. Our experience highlights the importance of careful preoperative investigation of the exact cause of the compression. If ALSA is the culprit, ALSA transection, a less invasive yet radical approach, appears to be a better option.

Asymptomatic KD, with a reported growth rate of 1.45±0.39 mm/year, may lead to subsequent enlargement and occurrence of compressive symptoms or even more fatal complications including aortic dissection and rupture.^8)^ Reported rates of dissection and rupture differ greatly from 0% to up to 50%.^1)^ KD’s high prevalence for cystic medial necrosis may at least partly explain its vulnerability to other vascular complications.^6)^ Such complications are not only life-threatening but also require highly invasive surgical treatments, and despite TEVAR’s inefficiency for compressive symptoms, it is reportedly effective for preventing dissection and rupture.^1,3,7,9)^ Although it is reasonable to conservatively manage asymptomatic KD, early endovascular treatment may be a viable option for patients with indicative lesions.

Both TEVAR and the FET technique are options available with regard to occluding KD orifice. Steep transition from the arch to the descending aorta, a common anatomy for patients with KD, often provides an inappropriate proximal landing zone for TEVAR.^7)^ In such cases, the FET technique with TAR can help overcome the unfavorable anatomy and concomitantly enables the anatomical reconstruction of the arch branches.

Conversely, the LSA reconstruction is debatable. Although its omission appears to be of less consequence in children, instances of limb ischemia and subclavian steal syndrome are reported in both children and adults.^4,5)^ We therefore believe that it should be routinely performed.

## Conclusion

A patient with symptomatic KD, RAA, and ALSA was successfully treated with a hybrid approach comprising ALSA transection, LSA reconstruction, TAR, and FET technique through median sternotomy. This case highlights a potential new treatment option, as well as the importance of preoperative investigation for the actual cause of compressive symptoms, as it greatly affects the surgical technique required.
